# Gene expression microarray analysis of the sciatic nerve of mice with diabetic neuropathy

**DOI:** 10.3892/ijmm.2014.2011

**Published:** 2014-11-26

**Authors:** LEI ZHANG, SHEN QU, AIBIN LIANG, HONG JIANG, HAO WANG

**Affiliations:** 1 Department of Special Needs Medical Branch, Shanghai Tongji Hospital, School of Medicine, Tongji University, Shanghai 200065, P.R. China; 2 Department of Endocrinology, Shanghai Tenth People’s Hospital, School of Medicine, Tongji University, Shanghai 200072, P.R. China; 3 Departments of Hematology, Tongji University, Shanghai 200065, P.R. China; 4 Radiology, Shanghai Tongji Hospital, School of Medicine, Tongji University, Shanghai 200065, P.R. China

**Keywords:** diabetic nephropathy, streptozotocin, rosiglitazone, target genes

## Abstract

The present study aimed to explore novel target genes that regulate the development of diabetic neuropathy (DN) by analyzing gene expression profiles in the sciatic nerve of infected mice. The GSE11343 microarray dataset, which was downloaded from Gene Expression Omnibus, included data on 4 control samples and 5 samples from mice with diabetes induced by streptozotocin (STZ), 5 samples from normal mice treated with rosiglitazone (Rosi) and 5 samples from mice with diabetes induced by STZ and treated with Rosi. Differentially expressed genes (DEGs) between the different groups were identified using the substitution augmentation modification redefinition (SAMR) model. The Gene Ontology (GO) term and Kyoto Encyclopedia of Genes and Genomes (KEGG) pathway enrichment analyses were performed using the Database for Annotation, Visualization and Integrated Discovery (DAVID). Regulatory and protein-protein interaction networks were searched using BioCarta and STRING, respectively. The protein structures of potential regulatory genes were predicted using the SYBYL program. Compared with the controls, 1,384 DEGs were identified in the mice with STZ-induced diabetes and 7 DEGs were identified in the mice treated with Rosi. There were 518 DEGs identified between the mice in the STZ + Rosi and STZ groups. We identified 45 GO items, and the calmodulin nerve phosphatase and chemokine signaling pathways were identified as the main pathways. Three genes [myristoylated alanine-rich protein kinase C substrate (*Marcks*), GLI pathogenesis-related 2 (*Glipr2*) and centrosomal protein 170 kDa (*Cep170*)] were found to be co-regulated by both STZ and Rosi, the protein structure of which was predicted and certain binding activity to Rosi was docked. Our study demonstrates that the *Marcks*, *Glipr2* and *Cep170* genes may be underlying drug targets in the treatment of DN.

## Introduction

Diabetic neuropathy (DN), a frequent chronic complication of diabetes (type I and II) (in approximately 60 to 75% of cases) (1), is a major cause of morbidity and mortality, potentially affecting the distal sensory, motor and autonomic nerves (2). DN typically manifests as autonomic dysfunction with postural hypotension, fainting, diarrhea and peripheral neuropathy with the loss of the sensation of pain and temperature followed by a pattern suggestive of a length-dependent degeneration of nerve fibers (3). The accurate and timely detection, characterization and quantification of DN are critical for the identification of patients at risk, the estimation of expected deterioration, the monitoring of progression, the assessment of novel therapies, and, ultimately, for the reduction of the incidence and cost of treatment for this disease. Current treatments for DN provide symptomatic relief rather than ameliorating disease progression (4). A previous clinical study on type II diabetes demonstrated that the insulin sensitizer rosiglitazone (Rosi) (Avandia), a synthetic agonist of peroxisome proliferator-activated receptor-γ (PPAR-γ) that is used to improve insulin resistance, is a potential drug for the treatment of DN (5). Another study however, demonstrated the undesirable effects of Rosi on the cardiovascular system. These findings suggested that treatment with Rosi may be harmful and should be used with caution in cardiovascular patients (6). Considering the fact that the etiology and pathogenesis of DN are not entirely understood, multicenter trials on the pathogenic mechanisms of DN are in urgently required. Moreover, there is an urgent need for the development of novel drugs with long-term effects on DN.

Streptozotocin (STZ), which selectively destroys pancreatic β cells, rapidly induces diabetes in mammals in a model of insulin-dependent diabetes (7). Gabapentin, pregabalin, amitriptyline, mexiletine and morphine, but not diclofenac, inhibit allodynia in a rodent model of STZ-induced DN, suggesting that the STZ-induced model of DN is suitable for evaluating the clinical potential of compounds for the treatment of painful DN (8).

In the present study, by microarray data analysis, differentially expressed genes (DEGs) were screened between a control (healthy) and a group of mice with STZ-induced diabetes, a control and a group of mice treated with Rosi, as well as between a group of mice with STZ-induced diabetes and mice with STZ-induced diabetes treated with Rosi. Based on functional annotation clustering analysis, processes significantly associated with this disease were identified in order to further elucidate the pathogenic mechanisms of this disese. Genes co-regulated by both STZ and Rosi were identified and the protein structure of the target genes was predicted. Proteins encoded by the identified genes are potential targets in the treatment of DN.

## Materials and methods

### Data source

All the microarray data were downloaded from the Gene Expression Omnibus database under the accession number GSE11343 (9), which included data on 4 control samples (GSM286159, GSM286160, GSM286163 and GSM286165), 5 samples from mice with diabetes induced by streptozotocin (STZ) (GSM286169, GSM286173, GSM286176, GSM286178 and GSM286430), 5 samples from normal (healthy) mice treated with Rosi (GSM286431, GSM286432, GSM286433, GSM286434 and GSM286436) and 5 samples from mice with diabetes induced by STZ and treated with Rosi (GSM286437, GSM286438, GSM286591, GSM286592 and GSM286593). The data contained expression profiles of 45,101 probes in total. The annotation platform was GPL1261 [Mouse430_2] Affymetrix Mouse Genome 430 2.0 Array.

### Identification of DEGs

Using an Affy pack with R programming language, 19 ChIP samples were pre-processed for statistical analysis. The significant analysis of microarray (SAM) method (10) was applied in order to identify the DEGs between the control and the group with STZ-induced diabetes, the control and the Rosi-treated group, as well as between the group with STZ-induced diabetes and the group with STZ-induced diabetes treated with Rosi (STZ + Rosi group). DEGs with a fold change ≥2 and Q-value <0.05 were selected.

### Function analysis of DEGs

The screened DEGs may control and regulate the development of DN. We wished to identify the DEGs affected by the utilization of both STZ and Rosi, potential genes regulating the development of DN, as well as the signaling pathways these genes are involved in. Thus, Gene Ontology (GO) term and Kyoto Encyclopedia of Genes and Genomes (KEGG) pathway (11) analyses were performed using The Database for Annotation, Visualization and Integrated Discovery (DAVID) (12). For each GO term (13), the P-value of function clustering and the p-value following multiple detecting correction, such as Benjamini correction or false discovery rate (FDR) correction, were calculated in DAVID. Moreover, the regulatory network of DEGs and the protein-protein interaction (PPI) network of proteins encoded by the DEGs was searched using the BioCarta and STRING online tools (14), respectively.

### Prediction of crystal structure of proteins

We searched the Protein Data Bank (PDB) database and universal protein resource (UniProt) database for the crystal structure of proteins encoded by genes (15,16). If there was no information available, the alignment of protein sequences was performed using BLAST software, as previously described (17) and we searched for the structure of homologous proteins in the PDB and UniProt databases.

### Prediction of novel potential drug targets

Rosi, belonging to the class of drugs known as thiazolidinediones, targets the PPAR-γ protein (18), and as previously reported, its application is limited by the side-effects of thiazolidinediones (19). Therefore, we further mined genes indirectly associated with PPAR-γ among potential regulatory genes as novel drug targets in the treatment of DN. The MOLCAD module in the SYBYL software (20) was used to predict the pockets of proteins and AutoDock software was adopted for docking (21).

## Results

### Identification of DEGs

**Figure 1:**
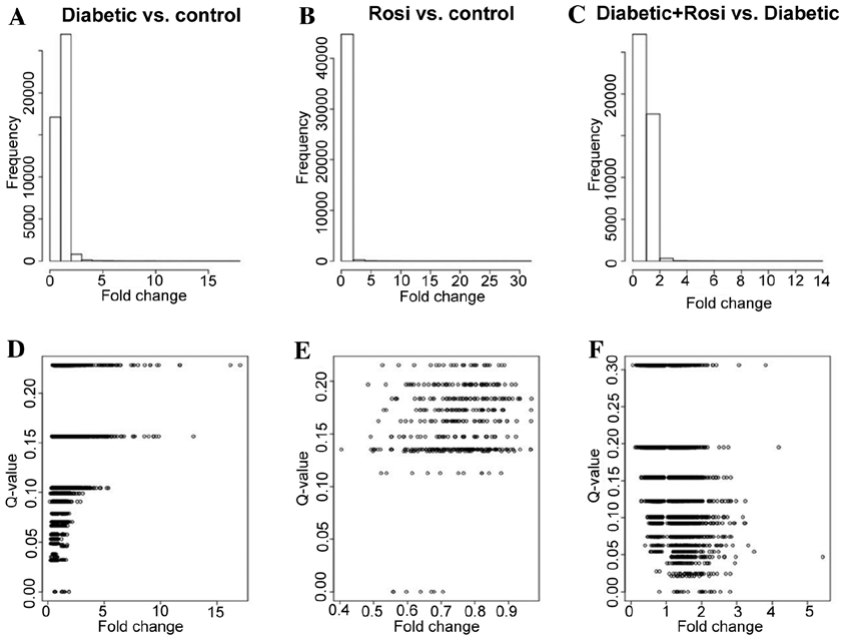
Distribution of variation of gene expression quantity following treatment with streptozotocin (STZ)/rosiglitazone (Rosi). (A) Histogram of gene expression variation of mice with diabetes induced by STZ. (B) Histogram of gene expression variation of mice treated with Rosi. (C) Histogram of gene expression variation of mice with STZ-induced diabetes and treated with Rosi compared with mice with STZ-induced diabetes. (D) Scatter diagram of gene expression variation and Q-value of upregulated/downregulated genes in mice with diabetes induced by STZ. (E) Scatter diagram of gene expression variation and Q-value of upregulated/downregulated genes in mice treated with Rosi. (F) Scatter diagram of gene expression variation and Q-value of upregulated/downregulated genes in mice with STZ-induced diabetes and treated with Rosi compared with mice with STZ-induced diabetes.

Compared with the control samples, there were 1,384 DEGs identified in the mice with STZ-induced diabetes with a fold change ≥2, including 85 DEGs with a Q-value <0.05; 7 DEGs were identified in the healthy mice treated with Rosi with a fold change ≥2, with no DEGs with a Q-value <0.05. There were 518 DEGs identified between the mice in the STZ + Rosi group and the mice with STZ-induced diabetes with a fold-change ≥2, including 41 DEGs with a Q-value <0.05 (Fig. 1).

**Figure 2:**
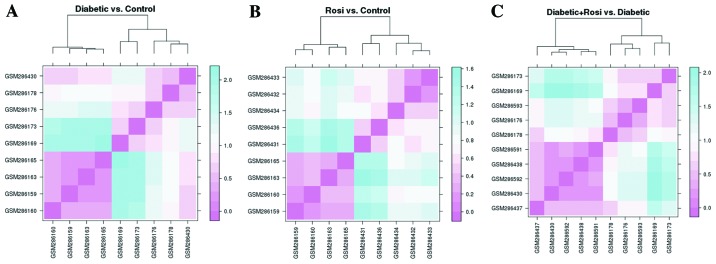
Clustering of corresponding ChIP samples from mice treated with streptozotocin (STZ)/rosiglitazone (Rosi). (A) Dendrogram of samples from mice with STZ-induced diabetes and control (no treatment); samples are represented by the log2 value of the 85 differential expressed genes identified. (B) Dendrogram of samples from mice treated with Rosi and control; samples are represented by log2 value of the 7 differential expressed genes identified. (C) Dendrogram of samples from mice with STZ-induced diabetes treated with Rosi and mice with STZ-induced diabetes; samples are represented by log2 value of the 41 differentially expressed genes identified.

The samples from the mice with STZ-induced diabetes and the healthy mice treated Rosi were effectively separated from the control samples (Fig. 2A and B), while the samples from the STZ + Rosi group could not be separated from those of the mice with STZ-induced diabetes completely (Fig. 2C). However, the samples from the mice in the STZ + Rosi group clustered in the first category (left), while the samples from the mice with STZ-induced diabetes gathered in the second category (right).

**Figure 3:**
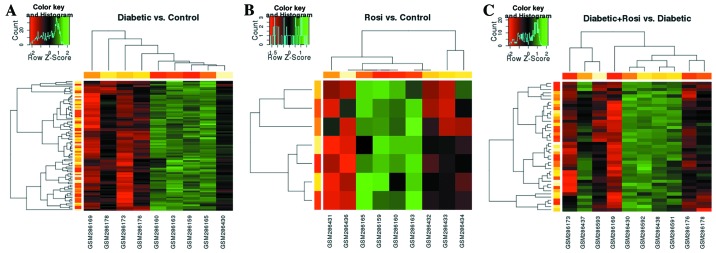
2D clustering of differentially expressed genes from samples of mice treated with streptozotocin (STZ)/rosiglitazone (Rosi). (A) 2D clustering of the 85 differentially expressed genes from the samples of mice with STZ-induced diabetes and control (no treatment). (B) 2D clustering of 7 differentially expressed genes from the samples samples from mice treated with Rosi and control. (C) 2D clustering of 41 differentially expressed genes of samples from mice with STZ-induced diabetes treated with Rosi and from mice with STZ-induced diabetes.

We further analyzed the DEGs in the samples following drug administration. As shown in Fig. 3A and B, following the administration of STZ or Rosi, the expression of all the DEGs was generically downregulated compared with the control samples. As shown in Fig. 3C, compared with the samples from the mice with STZ-induced diabetes, the expression of the DEGs in the samples from the STZ + Rosi group was mainly upregulated.

### Functional analysis of DEGs

**Figure 4:**
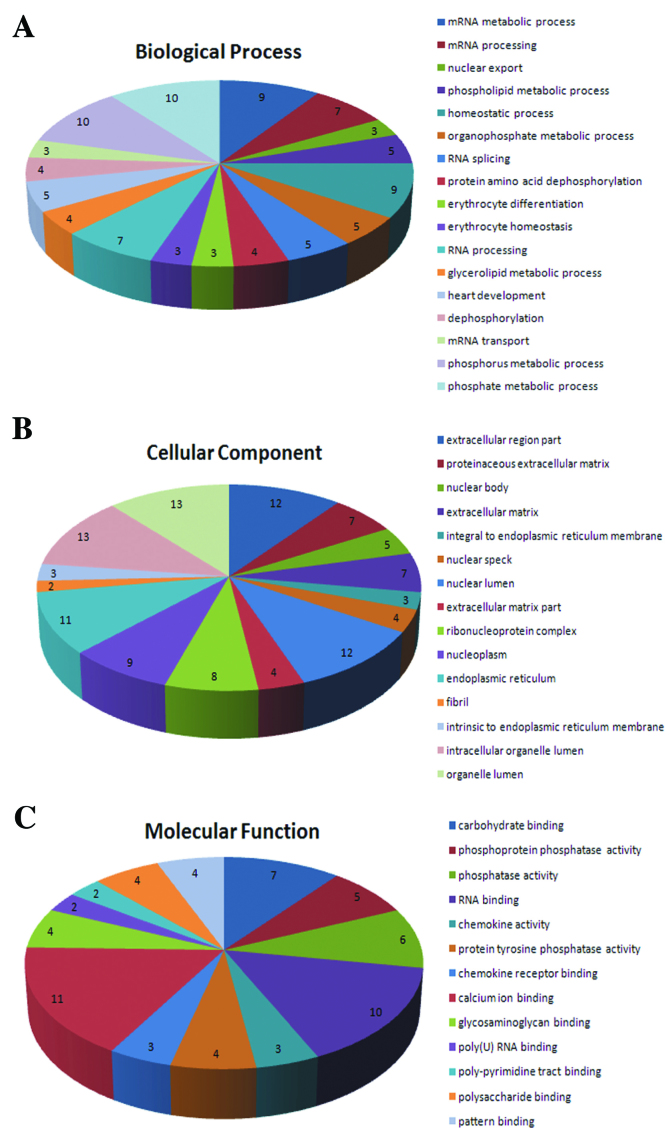
Gene Ontology (GO) functional annotation clustering analysis. (A) Significant biological processes identified; (B) significant subcellular location items identified; (C) significant molecular function items identified.

**Figure 5:**
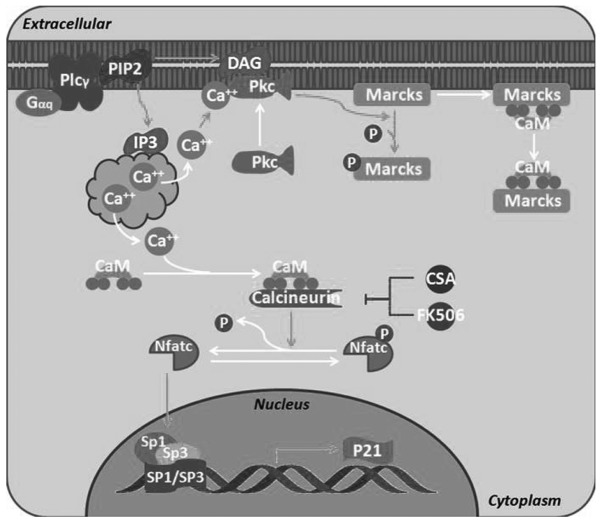
BioCarta analysis showed that the differentially expressed genes participated in the calmodulin nerve phosphatase pathways (calcineurin pathway: effects of calcineurin on keratinocyte differentiation); P=0.098.

After combining all the DEGs (fold change ≥2, Q-value <0.05), 123 DEGs were obtained. In the GO annotation analysis (P<0.05) of these genes, there were 17 biological process (BP) items, 15 cellular component (CC) items and 13 molecular function (MF) items identified (Fig. 4). The calmodulin nerve phosphatase pathway (P=0.098) (Fig. 5) and the chemokine signaling pathway (P=0.086) were identified as the most significant signaling pathways in enrichment analysis.

### Exploration of potential regulatory genes

By analyzing the 85 DEGs identitied in the mice with STZ-induced diabetes and the 41 DEGs identitied in the mice in the STZ + Rosi group, 3 genes were found to be co-regulated by STZ and Rosi; these were myristoylated alanine-rich protein kinase C substrate (*Marcks*), GLI pathogenesis-related 2 (*Glipr2*) and centrosomal protein 170 kDa (*Cep170*). As shown in Table I, these 3 genes were all downregulated in the mice with STZ-induced diabetes and upregulated in the mcie treated with Rosi. The recovery of gene expression illustrated that treatment with Rosi resulted in the upregulation of these genes. On the other hand, the downregulation of these genes following the administration of STZ suggests that the downregulation of these genes may contribute to the development of DN.

**Figure 6:**
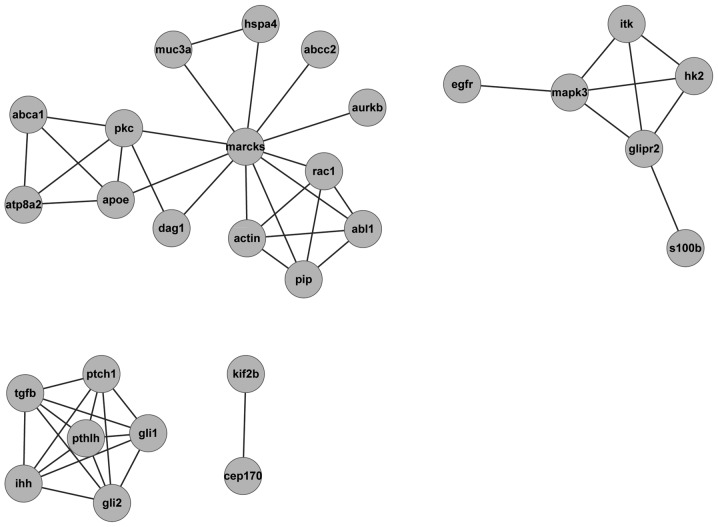
Protein-protein interaction network of proteins encoded by *Marcks*, *Glipr2* and *Cep170*.

In addition, the PPI network of the proteins encoded by these 3 genes was constructed using Cytoscape (Fig. 6). The protein regulating the upstream and downstream domains may regulate the 3 genes indirectly. For example, the actin protein connected with *Marcks* was related to axon guidance, which may be involved in the occurrence and development of DN.

### Prediction of novel potential targets

We searched the PDB crystal structure database in order to verify whether the proteins encoded by *Cep170*, *Marcks* and *Glipr2* were analyzed. It was found that Glipr2 was not analyzed in any type of crystal structure, while Cep170 and Marcks were only analyzed in the human crystal structure. The Cep170 and Marcks protein sequences from humans and mice were found in the UniProt database. The identity of first class sequences through BLAST comparison was 88 and 77%, with high homology. Considering that it has been previously reported that Rosi targets PPAR-γ (18), we selected the human crystal structure of PPAR-γ for comparison (identity was 62% compared with the mouse protein sequence).

**Figure 7:**
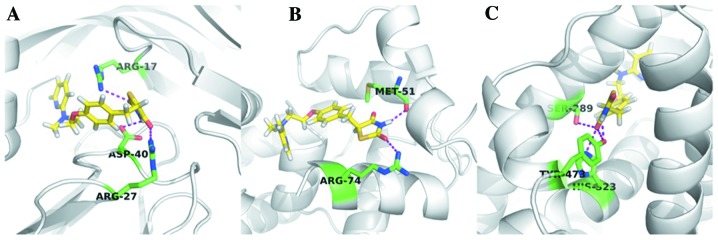
Docking of potential targets and rosiglitazone (Rosi) molecule. Yellow micromolecules represent Rosi, green residues are key residues forming hydrogen with Rosi, and the purple dotted line is the interaction of hydrogen. (A) Cep170 (4JON), (B) Marcks (1IWQ), (C) peroxisome proliferator-activated receptor-γ (PPAR-γ) (3V9V).

With the MOLCAD module using SYBYL software, the binding pockets of the 3 proteins were predicted (Table II). Using AutoDock, STZ and Rosi were docked to the crystal structures of Cep170 and Marcks, respectively. While docking Rosi to the crystal structure of PPAR-γ, a certain binding activity was found, as well as some key hydrogen bond interactions (Table II). The docking pattern of Rosi to the 3 proteins is shown in Fig. 7, which illustrates that Cep170 and Marcks may be the relevant targets of diabetes and DN, although the binding capacity of the 2 proteins was weaker than that of PPAR-γ.

## Discussion

DN is a common complication of type 1 and 2 diabetes, and affects approximately 20% of adult diabetic patients (22). At present, the pathology and pathogenesis of DN are not completely understood, and the majority of researchers consider that it is caused by multiple factors. In the present study, in GO annotation analysis of the identified DEGs, there were 17 BP items, 15 CC items and 13 MF items. The calmodulin nerve phosphatase pathways and chemokine signaling pathways were the main enriched signaling pathways identified. Additionally, in the study by Price *et al* (23) on the dorsal root ganglia of STZ-treated male Wistar rats, the authors examined diabetic peripheral nerves and found that the GO categorizing on glucose metabolism, oxidoreductase activity and manganese ion binding were significantly enriched for the regulated genes. The results of the current study are consistent with these findings. Price *et al* (23) also reported other enriched categories that we did not reproduce. This may either reflect inherent differences in gene expression between mice and rats, as well as between dorsal root ganglia and sciatic nerves; alternatively, this difference may also be due to the more stringent significance criteria in the current study. Therefore, our study provides the basis for further research on the pathogenic mechanisms of DN.

In this study, 3 DEGs (*Cep170*, *Marcks* and *Glipr2*) were found to be co-regulated by both STZ and Rosi. These 3 genes were downregulated in the mice with STZ-induced diabetes and their expression began to increase following treatment with Rosi. This result suggests that the downregulation of these genes may be responsible for the development of DN. A total of 85 DEGs regulated by STZ and 41 differentially expressed genes regulated by Rosi were verified, through the gene expression data analysis of 19 samples from mice with STZ-induced diabetes and those treated with Rosi, as well as from the mice in the STZ + Rosi group. The expression of these genes was increased following treatment with Rosi, which indicated that the proteins encoded by these 3 genes were potential targets in the treatment of DN. Guarguaglini *et al* (24) demonstrated that Cep170 is expressed throughout the cell cycle. It is associated with the centrosome in the interphase and with the spindle apparatus during mitosis. The overexpression of Cep170 in U2OS cells induced strong microtubule bundling, suggesting that Cep170 was able to bind microtubules with high affinity, and that Cep170 was involved in the regulation of microtubule dynamics (24). The dynamic properties of the cell cortex and its actin cytoskeleton determine important aspects of cell behavior and are a major target of cell regulation. The expression of Marcks has been found to be locally abundant and to correlate with morpho-genic processes and cell motility, and the protein accumulates at plasmalemmal rafts, where it is co-distributed with PI(4,5) P(2) and promotes its retention and clustering (25). DN is the most common cause of end-stage renal disease (ESRD) among type 2 diabetes mellitus patients (DM) in Malaysia (26). *Glipr2* has been identified as an upregulated gene in the study by Lokman *et al* (27). Huang *et al* (28) found that the expression of Glipr2 was elevated in the kidney tissue samples of patients with DN. However, further research on the function of *Cep170* in DN or other diabetic diseases were is required.

Rosi has been compared to metformin and glyburide as monotherapy in patients with recently diagnosed type 2 diabetes in A Diabetes Outcome Progression Trial (ADOPT) study. The results revealed a significantly lower risk reduction of failing monotherapy with Rosi compared to metformin and glyburide after 5 years of treatment (29).

In conclusion, in the present study, using a systems biology approach, we found significant changes in gene expression induced by treatment with STZ and Rosi. Furthermore, 3 differentially expressed genes were found to be potential novel targets in the treatment of DN. In addition, STZ and Rosi were tentatively docked to the binding pockets of these encoded proteins; the combined capacity of STZ and Rosi was weaker than that of PPAR-γ. If the molecular structure of Rosi could be modified, or if new molecules were found to target only Cep170 or Marcks rather than PPAR-γ, the side-effects of thiazolidinediones could be avoided. In brief, *Glipr2*, *Cep170* and *Marcks* are novel potential targets in the treatment of DN.

## Figures and Tables

**Table I:** Genes co-regulated by STZ and Rosi.

Gene name	STZ log2 (FC)^a^	Rosi log2 (FC)^b^	Function^c^
*Marcks*	−1.238	1.154	Marcks is the most prominent cellular substrate for protein kinase C (PKC); binds to calmodulin, actin and synapsin; inhibits F-actin cross-linking activity
*Glipr2*	−1.373	1.075	Unannotated
*Cep170*	−1.139	1.005	Plays a role in microtubule organization

a Log2 value of corresponding genes from mice with STZ-induced diabetes compared with the control (untreated) samples

b corresponding genes from mice with STZ-induced diabetes treated with Rosi compared with untreated mice with STZ-induced diabetes

c UniProtKB annotation function of the corresponding genes. FC, fold change; STZ, streptozotocin; Rosi, rosiglitazone.

**Table II:** Information of screened protein crystal structure and optimal combining capacity of butt joint.

Gene name	PDB ID^a^	Resolution (Å)^b^	No. of predicted pockets^c^	Ligand^d^	ePredicted optimal binding energy/affinity
*Cep170*	4JON	2.15	2	STZ	−4.83 kcal/mol (289.52 *μ*M)
				Rosi	−6.70 kcal/mol (12.29 *μ*M)
*Marcks*	1IWQ	2.00	2	STZ	−4.67 kcal/mol (378.72 *μ*M)
				Rosi	−6.01 kcal/mol (39.01 *μ*M)
*PPAR-γ*	3V9V	1.60	1	Rosi	−8.00 kcal/mol (1.37 nM)

a Protein Data Bank (PDB) ID number of corresponding crystal structure

b resolution ratio of the crystal structure

c number of binding pockets of identified inhibitor

d docking ligands

e combined capacity of ligands predicted by AutoDock and its corresponding protein. STZ, streptozotocin; Rosi, rosiglitazone; PPAR-γ, peroxisome proliferator-activated receptor-γ.
